# Das vestibuläre Labyrinth ist robuster als bisher gedacht – Erkenntnisse nach chirurgischer Entfernung intracochleärer Schwannome

**DOI:** 10.1007/s00106-022-01174-w

**Published:** 2022-04-28

**Authors:** Stefan K. Plontke, Torsten Rahne, Ian S. Curthoys, Bo Håkansson, Laura Fröhlich

**Affiliations:** 1 Universitätsklinik und Poliklinik für Hals-Nasen-Ohren-Heilkunde, Kopf- und Hals-Chirurgie, Martin-Luther-Universität Halle-Wittenberg, Ernst-Grube-Str. 40, 06120 Halle (Saale), Deutschland; 2 Vestibular Research Laboratory, School of Psychology, The University of Sydney, Sydney, NSW Australien; 3 Electrical Engineering, Chalmers University of Technology, Gothenburg, Schweden

Die Rezeptorsysteme für Hören und Gleichgewicht sind gemeinsam im knöchernen Labyrinth untergebracht und teilen sich die gleichen Flüssigkeiten. Bisher wurde angenommen, dass ein relevantes chirurgisches Trauma an einem der Rezeptorsysteme zu einem gewissen permanenten Verlust auch des anderen Rezeptorsystems führt. Anekdotische Berichte von einzelnen Patienten mit zumindest partiellem Erhalt der Cochleafunktion nach chirurgischer Zerstörung des vestibulären Labyrinths und vice versa haben dieses Prinzip infrage gestellt. Vor Kurzem konnten wir in einer großen Fallserie zeigen, dass nach relevantem chirurgischem Trauma der Cochlea im Rahmen der operativen Entfernung intracochleärer Schwannome die vestibulären Rezeptoren weiter normal funktionieren. Dies wurde mithilfe spezifischer, objektiver Funktionstests für alle fünf Gleichgewichtrezeptoren vor und nach der Operation nachgewiesen [1].

## Subtotale Cochleoektomie als unbeabsichtigtes chirurgisches Trauma-Modell

**Figure 1:**
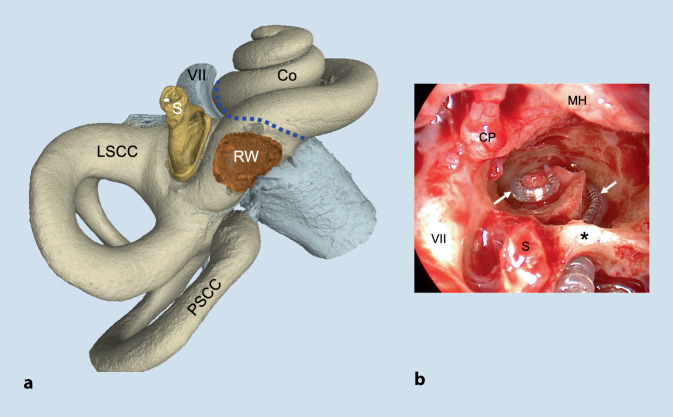

In den letzten Jahren konnte gezeigt werden, dass die chirurgische Therapie der sehr seltenen intracochleären Schwannome, welche sich oft mit einer Hörsturz- oder Menière-Symptomatik präsentieren, ein effektiver Weg für das Management dieser Tumoren darstellt. Unabhängig von der spezifischen Technik für die chirurgische Entfernung der Tumoren verursacht diese ein relevantes Trauma der Cochlea und eine Zerstörung der membranösen cochleären Labyrinthanteile. Die chirurgische Technik der partiellen oder subtotalen Cochleoektomie, welche wir üblicherweise zur Tumorentfernung anwenden, stellt ein – unbeabsichtigtes – Modell eines massiven Innenohrtraumas dar und ermöglicht neue Einblicke in die Funktion des vestibulären sensorischen Systems nach einem solchen Trauma (Abb. 1).

## Vestibuläre Funktionstests zeigen Erhalt der Rezeptorfunktion

Als Teil eines standardisierten Testprotokolls führten wir spezifische, objektive, vestibuläre Funktionstests vor und nach chirurgischem Trauma (subtotale Entfernung der Cochlea) für die Behandlung intracochleärer Tumoren bei 27 konsekutiven Patienten in unserem universitären Audiologischen, Hörimplantat- und Schädelbasis-Zentrum durch. Die vestibuläre Funktion wurde mithilfe kalorischer Testung (niederfrequente Antwort des lateralen Bogengangs), Testung des vestibulookulären Reflexes (Video-Kopfimpulstest, vKIT) aller drei Bogengänge sowie zervikaler und okulärer, vestibulär evozierter myogener Potenziale (cVEMP, Sacculus, und oVEMP, Utrikulus) gemessen. Die Otolithen-Funktion wurde aufgrund der postoperativen Schalleitungsschwerhörigkeit nach Entfernung des Ambosses mithilfe eines Knochenvibrationsstimulus beurteilt. Die prä- und postoperativen Verteilungen wurden mittels gepaarter *t-*Tests verglichen.

Die statistische Analyse zeigte keinen signifikanten Unterschied zwischen prä- und postoperativen Messwerten für alle Tests der fünf vestibulären Rezeptoren. Die Anzahl und Richtung des Spontannystagmus änderte sich nicht zwischen präoperativer und postoperativer Situation. Bei der Mehrheit der Patienten fand sich eine im Vergleich zur präoperativen Messung etwa gleiche oder verbesserte Antwort des lateralen Bogengangs nach kalorischer Reizung. Nur zwei Patienten zeigten in diesem Test eine Verschlechterung. Der vKIT-Gain blieb bei den meisten Patienten im Normbereich. Ein Patient verbesserte sich leicht, aber der Gain blieb bei den präoperativ bereits pathologischen Messwerten. Zwei Patienten verschlechterten sich im vKIT-Gain. In der anterioren und posterioren Ebene verschlechtern sich drei bzw. zwei Patienten postoperativ. Zwei Patienten verbesserten sich in beiden Ebenen leicht, aber der Gain blieb im pathologischen Bereich; ein Patient verbesserte sich. Bei den meisten Patienten zeigte sich postoperativ eine normale Otolithenfunktion (Sacculus und Utrikulus). Die Hörrehabilitation mittels Cochleaimplantat (CI) war erfolgreich, und das Einsilberverstehen bei einem Sprachpegel von 65 dB SPL verbesserte sich innerhalb des Beobachtungszeitraumes von zwölf Monaten kontinuierlich [1].

## Verschiedene Hypothesen für die Robustheit des vestibulären Labyrinths

Auch wenn die Mechanismen und Ursachen nicht genau bekannt sind, scheinen anatomische, physiologische und chirurgische Aspekte für die beschriebenen Beobachtungen ursächlich zu sein.

Erstens scheint die Anatomie günstig im Hinblick auf eine solche traumatische Intervention. So ist der Verbindungsgang zwischen den endolymphatischen Räumen der Cochlea und des vestibulären Labyrinths – der Ductus reuniens – sehr dünn (minimaler Durchmesser < 2 mm) und verschließt sich möglicherweise nach subtotaler Entfernung der Cochlea.

Zweitens sind offensichtlich ausreichend Endolymphe generierende Zellen im vestibulären Labyrinth vorhanden, sodass die vestibulären Rezeptoren funktionsfähig bleiben. Hauptsächlich sind dies die vestibulären „dark cells“ und die subepithelialen Melanozyten, die funktionell vergleichbar mit den Marginal- und Intermediärzellen der Stria vascularis der Cochlea sind. Somit kann die endolymphatische Kalium-Homöostase über eine feine Balance zwischen Sekretion und Absorption durch die epithelialen Zellen erhalten werden, welche auch die Voraussetzung für die Mechanotransduktion in den sensorischen Haarsinneszellen darstellt.

Letztlich spielt auch die chirurgische Technik eine Rolle. Diese ist charakterisiert durch extreme Vorsicht, Vermeidung von Saugung in der Nähe des Vestibulums, Arbeiten „unter Wasser“ so oft wie möglich und Verwendung einer Spülflüssigkeit, die künstlicher Perilymphe ähnlich ist. Auch das sorgfältige Abstopfen der Verbindung zwischen Cochlea und Vestibulum in der Region des Ductus reuniens scheint zum Erfolg beizutragen.

## Fazit für die Praxis

- Die Beobachtung, dass es nicht nur prinzipiell möglich ist, die vestibuläre Funktion auch nach subtotaler Cochleoektomie zu erhalten, sondern dass diese in den meisten Fällen erhalten werden kann, stellt übliche Konzepte negativer Effekte einer Chirurgie an der Cochlea auf die vestibuläre Funktion infrage.
- Diese Beobachtung könnte zukünftige Indikationen und Techniken der Chirurgie der Cochlea nachhaltig beeinflussen.
- In Verbindung mit der Beobachtung überdurchschnittlich guter Ergebnisse der Hörrehabilitation mit Cochleaimplantaten bei diesen Patienten bestärken diese Ergebnisse die Empfehlung für eine chirurgische Managementstrategie intracochleärer Schwannome und gleichzeitiger Cochleaimplantation im Gegensatz zu einer Radiotherapie oder einem „Wait-and-Test-and-Scan“-Vorgehen.
- Die anatomischen und physiologischen Hintergründe für die Robustheit des vestibulären Labyrinths gegenüber einem solchen chirurgischen Trauma müssen noch weiter erforscht werden.
